# A low‐grade astrocytoma in a sixteen‐year‐old boy with a 7q11.22 deletion

**DOI:** 10.1002/ccr3.1312

**Published:** 2017-12-22

**Authors:** Francoise S. van Kampen, Marianne E. Doornbos, Monique A. van Rijn, Yolande den Bever

**Affiliations:** ^1^ Department of Paediatrics Albert Schweitzer Hospital Dordrecht The Netherlands; ^2^ Department of Neurology Albert Schweitzer Hospital Dordrecht The Netherlands; ^3^ Department of Clinical Genetics Erasmus University Hospital Rotterdam The Netherlands

**Keywords:** AUTS2, brain tumor, neurologic, pediatric

## Abstract

We report a patient with developmental delay due to germline AUTS2 mutation who developed a low‐grade astrocytoma. While the contribution of this mutation to the pathogenesis of the tumor is not known at this time, a role of AUTS2 in deregulation of PRC1 can be a part in tumorigenesis of a brain tumor.

## Background

In this case report, we describe a first patient with a low‐grade astrocytoma and a deletion in the AUTS2 gene. Mutations in (parts of) the autism susceptibility candidate 2 (AUTS2) are described in case reports and further explored in animal models. The hypothesis is that the AUTS2 protein has an important role in axon guidance, dendrite elaboration, synaptogenesis or related aspects of neuronal development, but the exact function is not known. The phenotype has a wide range of features with intellectual disability, autism, and dysmorphic features. A role in tumorigenesis of brain tumors is not yet proved.

Low‐grade gliomas are the most common central nervous system tumors in childhood. Benign cerebellar astrocytomas are most prevalent, accounting for 15–25% of all central nervous system tumors 1, 2. Diffuse astrocytomas account for approximately 5% of all tumors in children aged 0–14 years 3. Malignant transformation in most low‐grade tumors is rare 2. First treatment of choice is complete resection of the tumor. After surgery, the long‐term results are good.

With time more is known about tumorigenesis. Mitogen‐activated protein kinase (MAPK) pathway activation drives the formation of most low‐grade gliomas (LGG). The most common mechanism of a MAPK pathway activation in LGG's is a tandem duplication on chromosome 7q34. In addition, deletions in 7q34 are described 4. Astrocytomas are occasionally described in Noonan, Turner, Lynch syndrome, and neurofibromatosis.

We present a patient with a low‐grade astrocytoma and a deletion of chromosome 7q11.22.

## Clinical Case Report

Our patient is the fifth child of nonconsanguineous Dutch parents. His eldest sister has ADHD and a mild intellectual disability (ID). His other sister and two brothers are healthy. The son of his father's sister has a coloboma of both eyes and a mild mental retardation.

Our patient was born term after normal pregnancy and no complications during and after delivery. He was diagnosed with a coloboma of the left eye in the neonatal period and at the age of 5 years, he was diagnosed with a mild intellectual disability (IQ 72) and he is following special education.

At the age of 12 years, he was evaluated for a small stature which was related to a late puberty. His puberty started at the age of fifteen and he reached a normal height.

In 2013, he had his first generalized tonic‐clonic epileptic seizure treated in the emergency department.

In the past 6 months, he had episodes of bilateral tinnitus with dizziness and difficulty speaking. During these episodes, duration of 30 sec and approximately three times a week, he turns his head to the left side and parents noticed an impairment of consciousness. After the episode, he complains of nausea and headache. These episodes were diagnosed as complex partial seizures.

On physical examination, we found mild dysmorphisms: hypotelorism, a broad forehead, a broad nose bridge, a full nose tip, and full lips. He has long feet and arms with a cubitus valgus.

**Figure 1:**
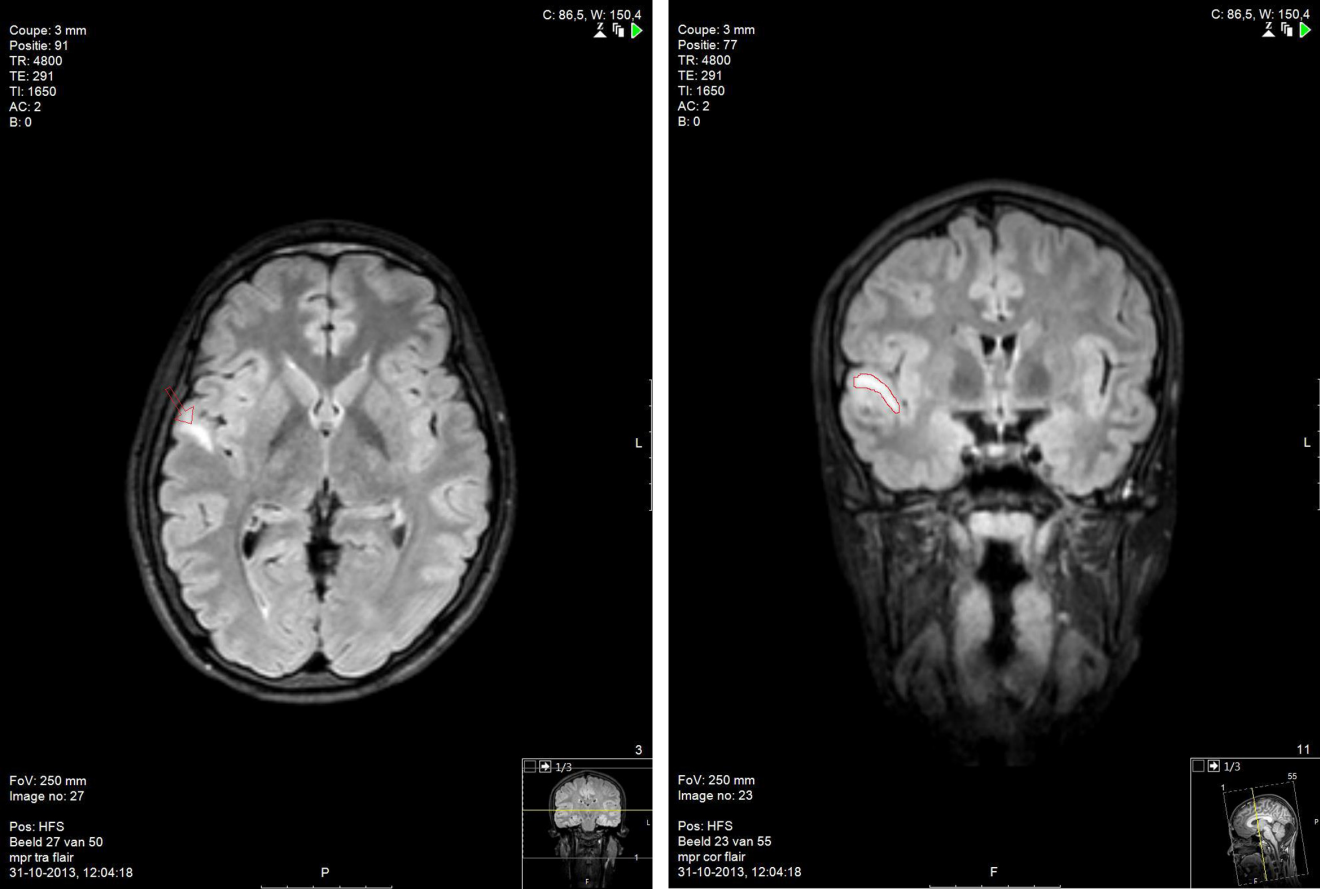
(A) Flair image with hyperintense lesion in the right temporal lobe (indicated by an arrow). (B) Flair image with hyperintense lesion in the right temporal lobe (indicated by a red line).

We performed an EEG and a MRI of the brain. The EEG showed mild diffuse slowing of the right hemisphere, but no epileptic activity. The MRI of the brain showed a hyperintense lesion on T2 and flair images in the right temporal lobe, with enhancement after admission of gadolinium. The radiological differential diagnosis included ganglioglioma, DNET, or low‐grade astrocytoma (Fig. 1).

**Figure 2:**
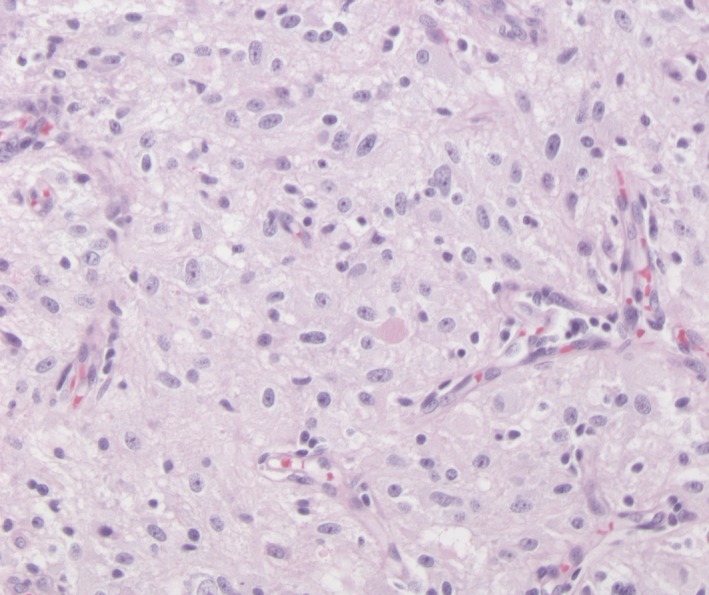
Photomicrograph of the tumour histology.

Consequently, he was operated in an academic hospital and the tumor was completely removed. Pathology shows a diffuse infiltrating low‐grade astrocytoma (Fig. 2).

No adjuvant therapy was given.

A MRI of the brain 6 months after surgery did not show residual tumor or recurrence of the tumor. After resection of the astrocytoma, the patient no longer suffered from seizures and a year after surgery, the antiepileptic drugs were successfully stopped. Follow‐up will be every 9 months until at least 5 years after the surgery.

## Results

The combination of a coloboma, a mild intellectual disability, a late puberty, mild dysmorphisms, and an epileptic seizure could well have a genetic cause, such as CHARGE syndrome. Tumors including a low‐grade astrocytoma can be part of various syndromes.

We performed a SNP‐array on total genomic DNA extracted from leucocytes. The Illumina Human Cyto 12‐SNP genotyping array was used in combination with the Illumina iScan Control and the Nexus Copy Number 7.0 software BioDiscovery El Segundo California, USA according to standard protocols of the manufacturer.

A deletion of 457 kb in band 7q11.22 (chr7: 68,910,981–69,367,635) was found. This loss includes 61 probes and a part of *AUTS2* including some exons (exon 2, 3 and 4).

In June 2015, we performed a whole‐exome sequencing also on total genomic DNA extracted from leucocytes for genes involved with ID, epilepsy, autism, and neuronal migration disorders. The WES did not show any causative mutations.

Pathology of the tumor shows a low‐grade diffuse astrocytoma, IDH‐wildtype, WHO grade 1 (corresponding to the 2016 WHO Classification system for CNS tumors).

Further genetic testing shows a point mutation in the BRAF v600e gene. This mutation is seen in <10% of all pilocytic astrocytomas.

## Discussion

Autism susceptibility candidate 2 (*AUTS2*) is located on chromosome 7q11.22 and spans 1.2 MB of the genomic DNA. *AUTS2* consist of 19 exons where the first half of the gene (exon 1–6) has relatively large introns and the second half (exons 7–19, C‐terminus) has smaller clustered introns. The exact function of *AUTS2* is unknown, but deletions in the *AUTS2* region gives a wide range of syndromic phenotypes, including intellectual disability, autism, microcephaly, short stature, hypotonia, cerebral palsy, and dysmorphic features. The phenotype can be subtle, and the severity of the syndrome is highly variable. Furthermore, the location and the type of deletion are important for the resulting phenotype, with a more severe phenotype with deletions in the C‐terminus 5. A recent study of Beunders et al. described 13 patients (including six adults) with *AUTS2* syndrome with unique pathogenic deletions scattered around the *AUTS2* locus and confirm a phenotype–genotype correlation 6.

The *AUTS2* protein also plays an important role in neurodevelopment. Earlier studies with knockdown *AUTS2* zebrafish shows a reduction in motor and sensory neurons in the spinal cord and developing neurons in regions that include the midbrain and cerebellum, which resulted in reduced movement and decreased response to touch 5, 7. Mouse studies show that there is expression of *AUTS2* in developing neurons in the frontal cortex and cerebellum. This suggests that *AUTS2* could be important for migration, axon guidance, dendrite elaboration, synaptogenesis, or related aspects of neuronal development 8.

The study of Hori et al. suggests that *AUTS2* may also have a cytoplasmic role in cytoskeletal regulation, in addition to its nuclear function, and indicate that cytoplasmic *AUTS2* is involved in cortical neuronal migration 9. They did not observe abnormal apoptosis or proliferation of cells in any of their *AUTS2* mutant mouse brains.

Gao et al. show a role for *AUTS2* in modulating the role of polycomb repressor complex 1 (PRC1) from a repressive function to transcription stimulation in the *AUTS2*‐PRC1 complex. In this way, *AUTS2* plays a role in regulation of neuronal gene expression through promoter association. Their hypothesis is that changes in *AUTS2* result in reduced neuronal gene expression trough the *AUTS2*‐PRC1 complex and can give different developmental defects 10. Polycomb group proteins play an important role in neurodevelopment, but deregulation can lead to oncogenesis. Deregulation of PRC1 plays a role in formation and progression of glioblastomas 11. BMI1 or polycomb group ring finger protein 4, which is part of PRC1, has a role in the suppression of p16^INK4a^ 12. Lack of expression of p16^INK4a^ next to a BRAF v600e mutation, gives less senescence and more tumor growth, where a BRAF v600e mutation alone is sufficient to promote only some aspects of oncogenic transformation 13. Probably changes in the *AUTS2* gene can result in deregulation of PRC1 through changes in the *AUTS2*‐PRC1 complex.

The possibility that this patient has two rare diseases which are not linked to each other can not be fully excluded, but may be less likely.

## Conclusions

This is the first case description of a patient with a mutation in the *AUTS2* gene and a brain tumor, in this case a low‐grade astrocytoma. Both the exact function of the *AUTS2* protein and the neurodevelopment of low‐grade gliomas are not yet known. There is a possible role for the *AUTS2* gene in the development of low‐grade gliomas via migration, axon guidance, dendrite elaboration, synaptogenesis, or related aspects of neuronal development. There may also be a role in tumorigenesis for the *AUTS2* protein by deregulation of PRC1.

## Authorship

FSK: was the main author and writer of the article. MED: was the doctor hereditary and congenital anomalies, diagnosed our patient with a syndromal problem, and the mentor of the main author. MAR: was the head practitioner of our patient and reviewer of the article. YB: was the clinical geneticist and performed all genetic test and checked our hypothesis.

## Conflict of Interest

The authors declare no conflict of interest.
